# In vivo neurochemical measurements in cerebral tissues using a droplet-based monitoring system

**DOI:** 10.1038/s41467-017-01419-1

**Published:** 2017-11-01

**Authors:** Guillaume Petit-Pierre, Philippe Colin, Estelle Laurer, Julien Déglon, Arnaud Bertsch, Aurélien Thomas, Bernard L. Schneider, Philippe Renaud

**Affiliations:** 1 Laboratory of Microsystems LMIS4, Ecole Polytechnique Fédérale de Lausanne (EPFL), Lausanne, Switzerland; 2 Brain Mind Institute, Ecole Polytechnique Fédérale de Lausanne (EPFL), Lausanne, Switzerland; 3 Unit of Toxicology, CURML, Lausanne University Hospital, Geneva University Hospitals, Lausanne-Geneva, Switzerland; 4 Faculty of Biology and Medicine, University of Lausanne, Lausanne, Switzerland

## Abstract

Direct collection of extracellular fluid (ECF) plays a central role in the monitoring of neurological disorders. Current approaches using microdialysis catheters are however drastically limited in term of temporal resolution. Here we show a functional in vivo validation of a droplet collection system included at the tip of a neural probe. The system comprises an advanced droplet formation mechanism which enables the collection of neurochemicals present in the brain ECF at high-temporal resolution. The probe was implanted in a rat brain and could successfully collect fluid samples organized in a train of droplets. A microfabricated target plate compatible with most of the surface-based detection methods was specifically developed for sample analysis. The time-resolved brain-fluid samples are analyzed using laser ablation inductively coupled plasma mass spectrometry (LA-ICP-MS). The results provide a time evolution picture of the cerebral tissues neurochemical composition for selected elements known for their involvement in neurodegenerative diseases.

## Abstract (web summary)

Current approaches studying the collection of extracellular fluid to monitor neurological disorders have temporal resolution limitations. Here the authors show functional in vivo validation of a droplet collection system included at the tip of a neural probe.

## Introduction

Neural probes, thanks to their reduced dimensions, present the ability to closely interface the central and peripheral nervous system. These complex devices can integrate microelectrodes which are both used to read and trigger neuron electrical activity and microchannels used for neurochemical stimulation or fluid sampling. Many evolutions of neural probes have taken place in the past 60 years, from the single platinum (Pt) microwire to complex systems which combine multiple microelectrodes with microchannels for local drug injection^1–5^. The implementation of these neurotechnologies has led to the development of efficient medical procedures such as deep brain stimulation (DBS) which significantly reduces the symptoms of Parkinson’s disease or cochlear stimulation which restores the functions of hearing impaired persons.

Microdialysis, a sampling technique used to monitor extracellular fluids (ECF), has become widely used in neuroscience^6, 7^. In this technique, a catheter inserted in a biological tissue is constantly perfused with a solution momently exchanging molecules with the ECF through a semi-permeable membrane. The perfusate is then collected and analyzed. The large acceptance of this method by the neuroscientist community is due to its relative simple operation and the statement that it preserves anatomical and functional integrity of the surrounding tissues^8^. However, microdialysis catheters have several limitations, starting with a relatively large exchange membrane area (diameter of 400 μm, length of 1−4 mm) limiting spatial resolution^9–11^. They only permit limited mass transfer with the ECF, since the molecules of interest need to cross a membrane by passive diffusion from the tissues to the perfusate^12^. Similarly, mass transport is limited by the size exclusion of the membrane which prevents large molecules such as proteins to be collected^13^. Besides the limited spatial resolution and associated tissue damages, the temporal resolution of microdialysis probe is usually limited to 10−20 min by the perfusion rate (typically 1 μL/min) and the associated analysis method^8^. With such a limitation, the method is probably only suited for observing long term kinetics of pharmacological agents and their effects in the brain^14^.

In contrast to microdialysis, push-pull probes are systems where the perfused solution directly contacts the brain tissues at the tip of the catheter before being recollected. Miniaturized low-flow (10−50 nL min^−1^) push-pull sampling probes were demonstrated to maintain a low amount of tissue damages in implanted tissues^15, 16^. Additionally, a major advantage of this approach is to provide a high collection efficiency compared to the microdialysis approach which is capital for sampling low concentration neurochemicals. This is mainly enabled by the direct contact of the perfusate with the ECF medium and the resulting fast diffusion occurring toward the collected solution, in contrast to the slow diffusion through a microdialysis membrane^15^. Although this approach is very promising since it could enable the rapid sampling of molecules of interest, Taylor dispersion which tends to spread the distribution of solute in a liquid flowing through a tube will distort the time-history of the sampling^17^. An answer to the dispersion issue during transport can be found in the implementation of segmented flows. We recently presented an approach which allows the collection of droplets with a high resolution (under the second). These droplets are separated by oily phases which maintain the temporal resolution defined by the lapse of time between two of them^18^. When applied to brain microsampling, the main challenge of this approach remains the analysis of the samples confined in low-volume, low-concentrated and non-purified samples surrounded by an oily carrier phase.

Surface sample-based mass spectrometry (MS) are powerful methods characterized by a high sensitivity to solid samples and limited preparation steps. The configuration of the reading zone (sample target) is often associated with an *xy*-stage which makes it compatible with various samples configuration including a set of droplets distributed at known position. Inductively coupled mass spectrometry (ICP-MS) is well known for the quantification of elements in biological samples at the trace and ultratrace level (<1 p.p.m.)^19^. The combination of a laser ablation (LA) system with ICP-MS provides a spatial dimension to the analysis. It has been recently used to determine the elements distribution (mapping or imaging) in thin-tissue sections of brain (20−30 μm thick) which contributed to the understanding of the biological processes involved in this region^20–23^. In particular, neurological studies focusing on monitoring the changes in concentration of Mn, Fe, Cu, and Zn occurring in Parkinson’s diseased (PD) rats were recently performed^24–26^. However, the analysis based on thin-tissue brain sections require the killing of the animal and only reflects the elements image of a tissue at a given point in time, which restricts the analysis to rather static observations.

In this work, we present a method for in vivo droplet-based sample collection combined with a specifically designed analytical platform. This unique combination permits the in vivo sampling of biological events with a high recovery efficiency as well as the analysis of their accompanying neurochemistry. The droplet collection system gives access to an additional dimension, the time evolution of the neurochemicals captured at a precise location in the brain. The droplets, stored in a capillary, constitute a spatio-temporal pattern reflecting the extracted liquid composition overtime. During the analysis the droplets separation and order must be preserved in order to maintain the sampling time-history. We solved this challenge by the development of a specific parylene base plate on which the droplet samples can be easily distributed and isolated at known positions. This platform is potentially compatible with multiple analysis systems using planar sample configuration as target. LA-ICP-MS was used to analyze the samples composition and enabled to determine precise trace element concentration of the droplets. This work set-up a bridge between neural probe technologies, advanced analysis methods and neuroscience. It provides opportunities for the neuroscientists with the perspective to study electrical stimulation on tissues and its related neurochemical response.

## Results

### Acute neurochemicals sampling

**Fig. 1:**
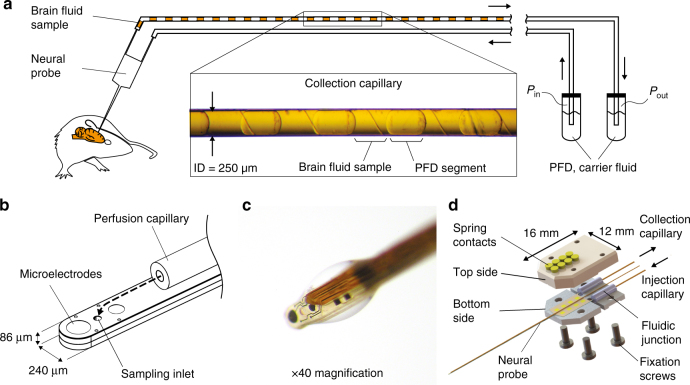
Neural probe system including technical details regarding the perfusion and the interface unit. **a** The brain-fluid samples are extracted using the neural probe and collected in a capillary. The samples are organized in a droplet train separated by Perfluoromethyldecalin (PFD), a non-aqueous phase. The system is controlled by two pressure pumps applying the pressure *P*_in_ and *P*_out_ to two reservoirs filled with PFD. The regularly spaced diagonal patterns on the collection capillary are manufacturing defects of the tubing outer layer. **b** The perfusion capillary is situated in close proximity to the main elements included on the neural probe distal tip. The dashed line shows the shortest path of the injected perfusate before being recollected in the sampling inlet (push-pull function). **c** A drop of saline solution is released via the perfusion capillary demonstrating the functionality of the system. The droplet wets the surface of the probe including the sampling inlet. **d** 3D exploded view of the interface unit comprising the neural probe clamped between the bottom and the top parts

**Fig. 2:**
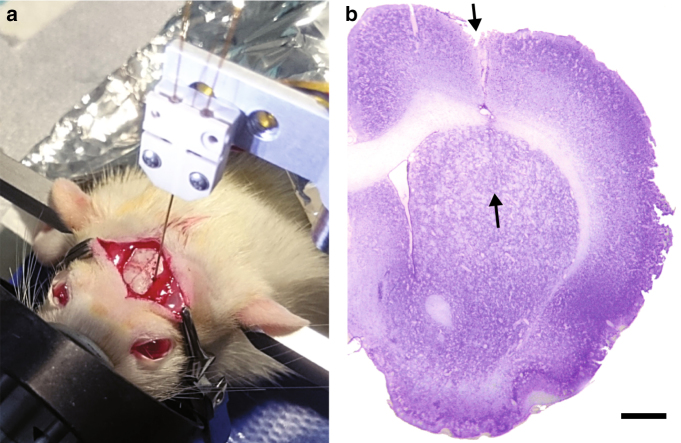
Surgical set-up during left brain hemisphere implantation on a rat. **a** The neural probe is maintained in the interface unit which is fixed on the stereotactic arm. **b** Stained left brain hemisphere after implantation of the neural probe. The black arrows represent the entry point and the end of the probe tip when inserted in the cerebral tissues (scale bar = 1 mm)

Acute neurosampling in rat brain tissues using an advanced droplet collection system included within a polymer-based probe was successfully demonstrated. Figure 1a shows a portion of a collection capillary filled with brain-fluid samples separated by PFD segments. We observed a regular spacing between the samples while the volume of each sample is similar with an average of 17.9 ± 1.8 nL (mean ± SD, *n* = 10). Interestingly, a change of the capillary inner surface properties occurred. Indeed, prior to use, the inner surface of the collection capillary was rendered hydrophobic using a silanization process. In preliminary experiments (ex vivo) consisting in the droplet sampling of deionized water with our system, the PFD segments showed high affinities with the inner surface of the capillary as expected. In in vivo conditions, after the collection of the brain-fluid samples, we noticed the contact angle between the glass surface and the PFD was rather 135 ± 5° which confirmed the inner surface of the capillary returned to a hydrophilic state. Supplementary Fig. 4 compares the PFD-liquid interface angle in these two situations and confirms a change of surface properties occurred. This rapid change is probably due to the binding of proteins against the inner capillary glass surface. Since the only liquid injected at the sampling spot is a saline solution (0.9% NaCl), this also proves qualitatively that the collected liquid samples captured molecules from the ECF. Figure 2a shows the surgical set-up during a measurement session. The neural probe is maintained in the interface unit which is fixed to the stereotactic-guided arm and can therefore be precisely implanted in the targeted brain area (striatum). Figure 2b shows a coronal slice which comprises the probe track in the tissues. This cross-section was specifically chosen among the others as it presents a maximum of damages to the tissues. The entry point as well as the end of the probe course can be identified in the upper brain structures (cortex region) and in the deeper region (top and bottom arrows, respectively). Nevertheless the probe track is difficult to identify, more particularly in the deeper region (striatum) were the impact on tissues is hardly distinguishable. The probe tip location is free of any void which confirms that no cavities are created by the perfusion and that a tight link between the probe and the tissues probably occurs.

### Analysis of in vivo samples

**Fig. 3:**
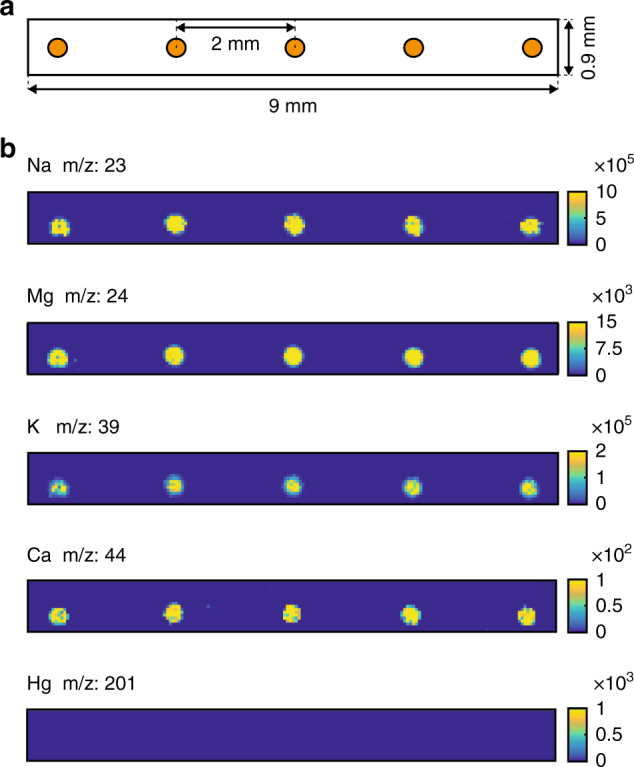
Brain-fluid samples imaging after LA-ICP-MS analysis. **a** Samples configuration prior to analysis. The parylene base plate includes five brain-fluid samples (orange spots) distributed along the substrate. **b** Relative abundance (a.u.) of the elements Na, Mg, K, Ca, and Hg (negative control) found in the brain-fluid samples

After the sampling process was completed, the brain-fluid samples were distributed on the parylene base plate. LA-ICP-MS mapping was performed on this substrate in order to detect the presence of elements contained in the brain-fluid samples. Figure 3a describes the location of the samples on the parylene base plate prior to analysis. The charts in Fig. 3b show the relative abundance (arbitrary unit) of selected elements as a function of the spatial positioning. The spatial resolution reached with the implemented analysis method allows to clearly identify each brain-fluid sample over the parylene base plate. We observe a strong signal for sodium (Na), magnesium (Mg), potassium (K), and calcium (Ca) at the position where the brain-fluid samples were distributed. As expected the signal of K and Na is particularly high, these ions being the most abundant in the brain extracellular fluid^27^. The last chart of Fig. 3b shows the relative abundance of mercury (Hg) well known as a neurotoxic metal^28^. We clearly conclude that no trace of Hg is present in the brain-fluid samples which was expected and therefore constitutes a negative control. Additional characterization of the imaging method is provided in Supplementary Methods (Supplementary Fig. 1).

**Fig. 4:**
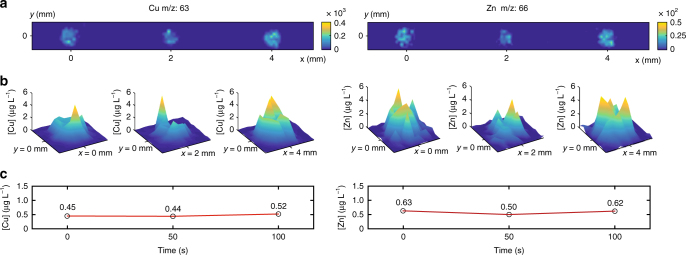
Time evolution of the trace metal concentration found in the brain-fluid samples reflecting the brain neurochemical state. **a** Trace metal relative abundance for Cu and Zn for three successive droplets. **b** 3D plots of Cu and Zn spatial concentration for each brain-fluid samples. The vertical axis is proportional to the concentration. **c** Temporal evolution of the concentration found in brain-fluid samples (sampling interval is 50 s)

The method of analysis implemented in this study is particularly powerful in the sense it is not limited to a single analyte but can be implemented on multiple elements with a single sample. Furthermore, it is compatible with the constitution of a time-based molecular concentration record, reflecting the brain neurochemichal composition overtime. In Fig. 4a, we present the relative abundance charts for copper (Cu) and zinc (Zn) taken from three successive brain-fluid samples (third degree linear interpolation was applied on intensity plots).

These metal elements are of particular interest for the neuroscientists since links with degenerative diseases have recently been highlighted. In particular Cu and Zn concentrations have been recently evaluated in Parkinson’s disease (PD) mouse models^24, 25^. Figure 4b shows 3D plots of metal concentration over each brain-fluid samples (the vertical axis is the relative concentration). The highest peak of each 3D plot is associated with the location of the highest color intensities found in Fig. 4a. Figure 4c shows the temporal evolution of the concentration found in each successive brain-fluid sample (mean spot value). This reflects the neurochemical composition of the implanted region overtime. The sampling interval was 50 s (sampling rate = 1.2 min^−1^). The metal concentration values reported are relatively stable in time which confirms the measurement method proposed here is consistent. Indeed, over this lap of time, we rather expect a constant biochemical composition of the cerebral tissues. The method validation is provided in Supplementary Methods (Supplementary Fig. 2, Supplementary Table 1).

Second column of Table 1 reports the mean concentration computed over the three brain-fluid samples (mean ± SD) for each metal elements. We found for Cu 0.47 ± 0.05 μg L^−1^ and for Zn 0.58 ± 0.07 μg L^−1^. We compared these values with recently reported basal concentration level of metal elements found in the rat hippocampus ECF measured using microdialysis^29, 30^. The mean Zn and Cu and concentration found are in good agreement with the reported values (0.51–5.22 and 0.77–2.48 μg L^−1^, respectively) although the mean copper concentration remained slightly under the range. Compared to microdialysis approaches, the measured values probably comprise metal ions originating from protein-bonds contained in the brain-fluid samples.

**Table 1:** Mean Zn and Cu concentration found in the 3 the brain-fluid samples (mean ± SD). The values are compared to basal concentration level found in the ECF of rat hippocampus.

**Element**	**Brain-fluid samples (μg L** ^**−1**^ **)**	**ECF rat (μg L** ^**−1**^ **)**
Zn	0.58 ± 0.03	0.50–5.22
Cu	0.47 ± 0.31	0.77–2.48

## Discussion

In this study, we demonstrated a method for in vivo acute neurosampling using an advanced neural probe system combined with a MS-based detection method. The associated analysis platform comprises a specifically developed parylene base plate which allows for an efficient brain-fluid sample isolation while maintaining the droplets order and avoiding cross-contamination. The flat sample configuration makes it potentially compatible with multiple surface-based analysis method. Although, we used ICP-MS in this work, the parylene base plate could easily be adapted and used in another spectroscopic-based equipment. For instance, MALDI-MS would be compatible and widen the range of analysis, allowing the identification and quantification of larger molecules, such as proteins or metabolites. The off-line measurement approach implemented in this work is interesting since it drastically simplifies the in vivo measurement phase. Disconnection of the collection capillary from the probe and storage at −20 °C of the droplet train was demonstrated to be particularly convenient. Distribution of the collected droplets on the parylene base plate even weeks after the implantation could be easily performed without affecting the samples. Another major advantage is that the brain-fluid samples are enclosed between PFD plugs in a glass capillary and thus fully isolated from oxidation which often occurs on molecules such as neurotransmitters (e.g., the Dopamine degradation to Dopamine o-quinone). A limit of the current method lies in the distribution of the droplets on the parylene base plate which is a manual operation. It introduces variability in the positioning of the droplets on the grid of holes. An option to overcome this limitation would be to automate the droplet distribution process by controlling the relative position of the capillary on the grid, possibly using a robotic arm. Precisely controlled positioning of the capillary, combined with a constant positive flow should result in a regular distribution of the droplets over the parylene base plate. During preliminary testing of the distribution process, we have noticed that even in the absence holes, it is possible to immobilize droplets and locally distribute solutes on the parylene film. Automating the distribution procedure could therefore allow to eliminate the need for performing the holes in the parylene layer, thus simplifying the fabrication process.

A major attribute of the neural probe is related to its unique droplet collection system. This sampling approach is compatible both with high-temporal resolution, but can also be operated in a slow, on-demand mode. Various applications can be envisaged to monitor biological events such as synaptic neurotransmission or changes in the basal chemical composition, which occur at very different time scales (milliseconds vs. hours). Although the diffusion time of small molecules such as metal ions from the ECF space to the sampling inlet of the neural probe will not be drastically faster compared to microdialysis approaches, improved temporal resolution for this specific situation will still occur in our system due to droplet segmentation. Indeed, the segmentation of the collected liquid at the distal tip avoids Taylor dispersion and further maintains the temporal history of the samples. This approach however requires the droplets to remain tightly separated. In the current device, the temporal resolution is defined at 50 s which is the lapse of time between the constitution of two consecutive droplets in the collection capillary. These droplets are formed by the merging of smaller droplets, the microchannel droplets. The merging occurs in an expansion zone where the neural probe microfluidic outlet meats the collection capillary. The merging is due to the difference of area between the probe microchannel cross-section (40 × 80 μm) and the inner diameter of the collection capillary (ID = 250 μm). Consequently, around 20 microchannel droplets, each of a volume of roughly 0.84 nL as specified elsewhere^18^, merge at this specific point until the constitution of a larger droplet, big enough to be transported by the carrier phase (PFD). This constitutes a loss of temporal resolution. A way to address this issue would be to reduce the fused silica capillaries dimension (e.g., ID = 50 μm) thus reducing the gap of area between both cross sections. This would however require to redesign another connector used at this fluidic junction and, possibly, the probe itself to facilitate the integration. As shown in Fig. 1a, the inner surface of the collection capillary, which is initially hydrophobic following silane gas treatment, becomes hydrophilic after the passage of the liquid sampled in the brain. This change in surface properties of the capillary inner wall may as well contribute to the contact between droplets. Improving the capillary wall hydrophobic properties could address this limitation. This could be achieved by the deposition of a stable hydrophobic film (1–2 μm) on the inner wall of the capillary.

As a first approach, during the in vivo experiment we perfused more saline (=2000 nL) in the brain than the volume retrieved by the collection system ($\cong$500 nL) in order to avoid excessive drying of the tissues in contact with the probe tip. It is not expected that the fluid excess ($\cong$1500 nL) negatively impacts the surrounding tissues. The absence of void space at the tip of the probe in the tissue section from Fig. 2b confirms this hypothesis. The excess of physiological solution is mainly absorbed by the neural tissues in a radial diffusion manner. Diffusion is the major mechanism driving the evacuation of excess fluid in the brain tissues^31, 32^. The brain contains around 20% (in volume) of ECF which facilitates this process^14^. A future improvement of this process will consist in perfusing the same amount of saline than the total retrieved brain-fluid volume.

The role in brain function of major cations, such as Na^+^, K^+^, Ca^+^, and Mg^2+^ have been thoroughly characterized. Although less studied, transition metal elements are also known to play critical roles in synaptic transmission, and might be implicated in neurological diseases^8, 33^. For instance, Fe is a cofactor for a variety of enzymes responsible for the synthesis of dopamine in synapses^8, 34, 35^, Cu is hypothesized to inhibit the voltage-gated calcium channel of synapses and is responsible for impaired neurotransmission when its concentration in too high^8, 36, 37^ while Zn^2+^ is involved in the release of the glutamate and *γ*-aminobutyric acid (GABA) release^8, 38–40^. Although our system is compatible to different types of surface-based analysis, LA-ICP-MS is a promising method for neuroscience applications since it enables to draw a picture of trace elements distribution in a brain section at a given time. Compared to the current approach consisting in mapping a brain tissue sections from an animal killed at a given point in time, the method proposed here is minimally invasive and allows for multiple recordings performed on a same animal. This is of particular interest when the focus of the study is linked to neurodegenerative studies like Parkinson’s (PD) or Alzheimer’s (AD) disease which develop over long time scale. Our approach doesn’t allow to perform multi-site chemical mapping but it gives access to a third dimension, the time evolution, at a very precise location recorded in the brain which can be of major interest when studying PD or AD. In particular the metabolic exchanges occurring between neurons and astrocytes is the first system to be impacted in neurodegenerative diseases. Therefore the recording of metabolites such as pyruvate and lactate with our neural probe would be of major interest in PD and AD research. The dynamic of these metabolic exchanges typically occur in a time range (few minutes) compatible with the demonstrated temporal resolution of our system^41, 42^. Dynamic neurochemical recording would also be highly beneficial in pharmacokinetic studies for the development of next generation drug therapies in PD and AD. In PD for instance, improving the response time of a drug treatment is a major challenge which has a direct impact on the therapeutic outcome^43^. Within this context, performing pharmacokinetic studies with our fast time-segmented recording approach would be of major interest. The pharmacodynamic response in the brain striatum of the drug Levodopa for PD typically occurs in tens of minutes which is compatible with the temporal resolution of our device^44, 45^.

In this study, we present the in vivo validation of a microscale droplet collection system included at the very distal tip of a neural probe. With this device, we successfully sampled neurochemicals present in the brain ECF of a rat. We developed a unique parylene base plate to fix and analyze our samples which is compatible with multiple surface-based detection approaches. LA-ICP-MS was used to analyze the brain-fluid samples. The results confirmed the presence of elements such as Na, Mg, K, and Ca which are known for their contribution in the regulation of biological processes at the synaptic level. Furthermore quantification (droplet by droplet) was demonstrated for the trace elements Cu and Zn known for playing a role in neurodegenerative disease development. The collected samples constituted a high resolution spatio-temporal pattern (droplets interval = 50 s) representative of the neurochemical state at the tip of the implanted probe. We foresee a strong potential for using this technology in combination with other surface-based detection methods thus further extending the molecular coverage. This tool constitutes an advancement toward the precise monitoring of time-dependent biological events in neuroscience.

## Methods

### Probe

A neural probe combining microelectrodes and a microfluidic channel system (monolithic integration) has been designed and assembled. The distal tip includes a T-junction droplet generator which enables the collection and the extraction of ECF-containing fluid plugs separated by an inert oil (perfuloromethyldecalin, PFD). The microfabrication approach integrates every active element of the distal tip (microelectrodes and sampling hole) on a same surface. A polyimide/SU-8 sandwich structure constitutes the base materials of the neural probe. The microelectrodes are made of Platinum (Pt) while the entire structure is built on the top of a sacrificial layer (Aluminum) dissolved when releasing the probe according to the method developed by Metz et al.^46^. Extensive explanation regarding the fabrication process as well as characterization of the system can be found in ref. ^18^. A fluidic channel was bonded (EPO-TEK 301-2FL, Epoxy Technology) on the same surface than the one containing all the active elements, its outlet being in close proximity to the sampling inlet (300 μm). It is constituted of a fused silica capillary (ID = 50 μm and OD = 196 μm) and is used for the perfusion of a low flow saline solution (0.9% NaCl) in the area where the microsampling occurs. The capillary is smaller than the width of the probe (240 μm) which makes it compliant with the system compactness and minimally invasive feature. Figure 1a shows schematically the neural probe device during collection of brain-fluid samples. The neural probe includes two microfluidic entries (proximal side) which can be operated separately via two pressure-driven reservoirs (Fluigent, module MFCS-MZ) filled with PFD. Two fused silica capillaries (ID = 250 μm, OD = 360 μm and length = 40 cm) connects the probe microfluidic entries with the reservoirs. When the sampling process is started, the collection capillary is filled with brain-fluid samples separated by PFD plugs while the injection capillary is filled with PFD only. With this system, one can apply a positive or negative pressure (relatively to local pressure) on the microchannels filled with PFD, thus controlling the droplet generation process occurring at the probe distal tip^18^. Figure 1b shows the distal tip of the neural probe including the main elements such as the sampling inlet, the microelectrodes and the perfusion capillary. The pathway of the injected perfusate before being recollected in the sampling inlet is represented by the dashed line. On Fig. 1c, a drop of saline solution is released via the same capillary demonstrating the functionality of the system. It can be noted that the drop efficiently wets the probe tip including the sampling inlet.

### Interface unit

Polymer-based neural probes are often difficult to interface because they are mechanically flexible and therefore difficult to manipulate or to connect to harder materials. We solved this issue by designing an interface unit particularly small and light allowing for a reliable connection to the electrical contacts as well as the fluidic microchannels of the probe. Figure 1d shows an exploded 3D view of the main elements of the interface unit in which the probe can be clamped after connecting the top and bottom part. Four fixation screws are used to connect the parts together while the electrical connection is made with gold spring contacts. The materials used to build the interface unit were chosen for their good biocompatibilities (peek, stainless steel, silicone, and gold) which makes the system compliant with in vivo experimentation.

### In vivo experiments

All experiment procedures were performed in accordance with the local animal care authorities. A Sprague Dawley female rat (Charles river laboratory) weighting 270 g was included in the experiment. The procedure was performed under Isoflurane anesthesia (4% at induction, 2% for maintenance) and included, prior to any surgical act, a preliminary analgesia by buprenorphine injected under the neck skin (Temgesic, 0.1 mg/kg). The subject was fixed in a stereotactic frame which allows the precise targeting of the brain striatum. Stereotactic coordinates were set to +0.5 mm anterior, +3.0 mm lateral and −5.0 mm ventral from bregma and the toothbar was set to −3.3 mm. First a craniotomy was performed on the subject using a drill; the external diameter of the hole being relatively small with 0.8 mm. Prior to implantation the capillaries and the probe microfluidic network were filled with PFD. Then the probe was implanted slowly in the tissues while injecting the saline solution through the perfusion capillary at a constant flow rate of 50 nL/min in order to avoid clogging. Five minutes after the target area was reached, the droplet sampling system was turned on. During this phase, a positive pressure *P*_in_ = 50 mbar and a negative pressure *P*_out_ = −350 mbar was applied (relatively to the local pressure) during 20 min. Ten seconds prior to starting the sampling, saline solution (0.9% NaCl) was injected at a flow rate of 100 nL min^1^ using the perfusion capillary. The saline perfusion was maintained during the entire sampling procedure. After the sampling, the probe was slowly retracted from the tissues. After the procedure, the rat brain was transcardially perfused with a 4 paraformaldehyde containing saline solution. 25 μm slices were cut from the brain in the region of the implantation using a Microtome (Leica biosystems) and placed in a sodium azide solution. The slices were first separated and stained (1 min in cresyl violet dye) and then rinsed with ethanol and toluol solvents. At the end of the experiment, the collection capillary in which the droplets were collected was disconnected from the probe and stored at −20 °C. Particular level of attention was provided to maximize the animal well-being along the in vivo experiment phase.

### Parylene base plate fabrication

One of the most important concepts of the neural probe presented in this work is to maintain a high-temporal resolution by keeping a spatial separation between each collected droplet. Taylor dispersion is avoided from the very beginning of the collection as the droplets are generated within the probe distal tip (no dead volume). As the probe includes an open sampling inlet (no membrane), the system temporal resolution is defined as the lapse of time between two consecutive droplets later analyzed. A focus of this work was therefore to maintain this separation as well as the correct order between droplets at every step of the analytical process. For this purpose, a parylene-based substrate was developed and built using microfabrication methods. A float glass wafer was first piranha cleaned, and then coated with a 2 μm parylene layer. An Excimer laser (Optek LSV3) was then used to drill 20 μm holes with a specific pattern on the parylene membrane. The laser parameters were adjusted in order to drill the holes until reaching the float glass wafer without altering its surface (power 2.5 kJ, attenuation 50%, 15 repetitions, repetition rate 50 Hz). Argon gas protection was used in order to limit redeposition of the burned waste on the substrate. After manufacturing the parylene base plate comprised a grid of holes equally spaced according to an optimized pattern which could efficiently capture aqueous droplets.

### Droplets distribution procedure

**Fig. 5:**
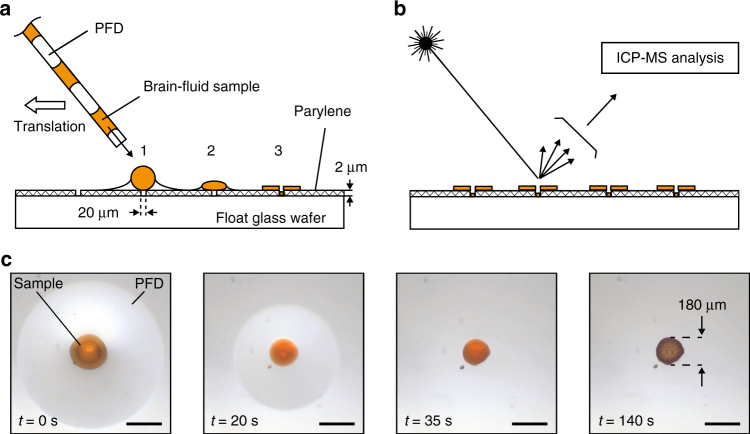
Droplet distribution procedure and analysis on the parylene base plate. **a** The brain-fluid samples are distributed on 20 μm drilled holes in a 2 μm thick parylene layer. The float glass wafer is hydrophilic and tends to fix the aqueous samples in position while the hydrophobic parylene tends to extract away the PFD oil. **b** After drying, the brain-fluid samples are laser ablated and analyzed using ICP-MS. **c** Drying sequence which shows the evolution of a red-colored water droplet and the adjacent PFD carrier phase (scale bar = 200 μm)

The developed parylene base plate described above enables the distribution of the droplets, one by one, on the parylene membrane thus avoiding cross-contamination or loss of order. As showed in Fig. 5a, the droplet distribution procedure consisted in slowly moving the collection capillary filled with brain-fluid samples parallel to the parylene surface while applying a positive flow of 0.02 μl/min using a syringe pump (Cetoni, Nemesys). A parylene base plate typically includes 1 row of 5 similar holes, the holes being 2 mm spaced (x direction) while the beginning and the end of the series are signaled by a cross shape (laser machined).

In order to validate this tailor-made parylene base plate and accompanying distribution method, a red-colored water droplet enclosed between 2 PFD plugs stored in a capillary was distributed over a parylene hole and monitored until complete drying. Figure 5c shows a sequence of images during which the drying process occurs (140 s, at room temperature under microscope light). The PFD (clear liquid) evaporates rapidly (35 s) while the red-colored droplet takes longer to dry (140 s). This is due to the high affinity of the PFD with the parylene membrane tending to spread the oil and increase the exchange surface while the contrary occurs for the water droplet. A slight recentering of the water sample over the parylene hole occurs during the first 20 s which is beneficial for the line by line analysis defined with the laser ablation system coupled to ICP-MS. The final diameter of the dried spot is 180 μm. Once dried, the samples are easier to manipulate and compatible with different surface-based analysis methods. Compatible MS-based approaches would typically comprise matrix assisted laser ionization mass spectrometry (MALDI-MS), secondary ion mass spectrometry (SIMS) or desorption electrospray ionization mass spectrometry (DESI-MS). In order to minimize any potential sample contamination, the sample distribution took place under a laminar flow in controlled environment and the plate was protected in dedicated closed carrier tray during its entire life cycle, from microfabrication to samples analysis.

### LA-ICP-MS

As a first approach, the measurements were performed using an inductively coupled quadrupole-based mass spectrometer (7700 series ICP, Agilent technologies) coupled to a solid state laser ablation system (NWR213, ESI). This equipment comprised a focused Nd:YAG system used to laser ablate the sample material. The resulting products were then transported by helium (carrier gas) into the inductively coupled plasma (ICP) which ionized the samples. The positively charged ions were then extracted from the plasma and analyzed in a quadrupole mass spectrometer (MS) which separated them with respect to their mass-to-charge ratio to be finally detected by the ion detector. This system is known for detecting metals and several non-metal elements at extremely low concentration (<1 p.p.m.) and is commonly used to perform cerebral bioimaging on brain tissue sections^20^. The parameters used to perform the analysis of the brain-fluid samples were defined as the following; laser energy density 5.2 J cm^−2^, energy output 17%, laser focus spot size 50 μm, laser pulse duration 20 ns, repetition rate 20 Hz, scanning speed 50 μm s^−1^, the MS acquisition rate 1 Hz and the carrier gas flow rate 800 ml min^−1^. Two-dimensional (2D) imaging was performed on the previously described parylene base plate after the distribution of the brain-fluid samples, as shown in Fig. 5b. All data were processed using Matlab and visualized using MSiReader^47^.

### Quantification

A standard calibration solution (Multi-element 2A, Agilent technologies) was used to generate the calibration curves employed for quantifying the metal elements found in the brain-fluid samples. Deionized water solutions with known concentration of metal standards (27 elements, 100 ng mL^−1^, 1 and 10 μg mL^−1^) were prepared. In order to mimic the final configuration of the in vivo collected samples, droplet trains of standards solution separated by PFD plugs were prepared using a flow focusing system driven by 2 syringe pumps (Cetoni, Nemesys). The droplets were collected in capillaries and then distributed over the parylene base plate. LA-ICP-MS analysis was performed on these samples and calibration curves were constituted considering the maximum concentration value averaged over five droplets.

### Data availability

The data generated and/or analyzed during the current study are available from the corresponding author on reasonable request.

## Electronic supplementary material

Supplementary Information
