# Supplementary material for: In vivo neurochemical measurements in cerebral tissues using a droplet-based monitoring system
Source: Nat Commun. 2017 Nov 1;8:1239. doi: 10.1038/s41467-017-01419-1 (PMC5665973; doi:10.1038/s41467-017-01419-1)
Supplement: Supplementary file 1 — Supplementary Information [file 41467_2017_1419_MOESM1_ESM.pdf]

Supplementary methods

Analysis method - initial characterization

In a first phase, the validity of the analysis method for the detection of samples collected from the brain was assessed as part of an *in vivo* pilot experiment. The conditions were mainly the same as described in section 2 (Materials and methods). The LA-ICP-MS parameters used during the analysis of the samples were modified. In order to speed up the analysis for this initial characterization the laser scanning speed was increased to $100\,\mu\mathrm{m\,s^{-1}}$ and the laser focus spot diameter was doubled to reach $100\,\mu\mathrm{m}$. A single laser ablation pass was performed over the dried brain-fluid spots. The probe driving parameters were slightly modified during *in vivo* sampling; P_{in} was set to $50\,\mathrm{mbar}$ and P_{out} to $-400\,\mathrm{mbar}$.

Supplementary Fig. 1 (a) shows the scanning area ($10\,\mathrm{mm}$ x $2\,\mathrm{mm}$) comprising 5 brain-fluid spots as well as 5 control spots which were laser ablated in 3 consecutive passes (space between 2 spots = $2\,\mathrm{mm}$, distance between 2 lines = $1\,\mathrm{mm}$). The first pass was performed over the 5 brain-fluid samples collected *in vivo* (upper section, orange spots), the second pass was set over an area covered with parylene only (middle section) and the third pass went over 5 control spots (bottom section, white spots) constituted of the *in vivo* perfused saline solution (0.9 % NaCl). Supplementary Fig. 1 (b) shows different charts of relative abundance for a selected set of elements (Na, Mg, K, Ca and Hg) detected in the scanning area as defined above. Strong signal intensity is observed for Na, Mg, K and Ca in the upper section, at the location where the brain-fluid samples were distributed. The chart of Na is of particular interest since the signal is also present over the bottom section of the scanning area, at the spots location of the perfused saline solution (0.9 % NaCl), which was expected. The absence of signal in the bottom section on the other charts confirms the concerned elements were collected from the tissues and were not present in the perfusion. This constitutes a first control. As expected, no trace of Hg is found in the brain-fluid samples as confirmed by the last chart which constitutes a negative control. No signal is observed in the parylene-covered region (second row of the laser trace, every charts) which confirms parylene does not interfere with the analysis. This constitutes a first blank control. Similarly, the regions in-between the aligned brain-fluid samples covered by PFD only (during the droplets distribution process) do not generate any signal which could interfere with the analyzed elements. This constitutes a second blank control.

We observe that the width of each droplet on the first line is variable, the second droplet signal being particularly large, the third being small. This probably results from a slight misalignment of the droplet dried spots with respect to the path of the laser beam as exposed in Supplementary Fig. 2. Based on the direct observation of the laser beam path during analysis, Supplementary Fig. 2 provides an example of misalignment which can occur. Supplementary Fig. 2 (a) shows the droplets (black) and the location of the parylene holes (white crosses). Supplementary Fig. 2 (b) shows the laser beam trace over the dried spots (laser focus diameter is $100\,\mu\mathrm{m}$) while Supplementary Fig. 2 (c) shows the equivalent droplet area "seen" by the laser and the detection equipment. Finally, the signal output relative intensities are represented on Supplementary Fig. 2 (d). Apart from the sample initial volume, the pixel widths depend on the speed of acquisition of the MS equipment as well as the speed of the laser beam (acquisition rate = 1 Hz, laser beam speed = $100\,\mu\mathrm{m\,s^{-1}}$)

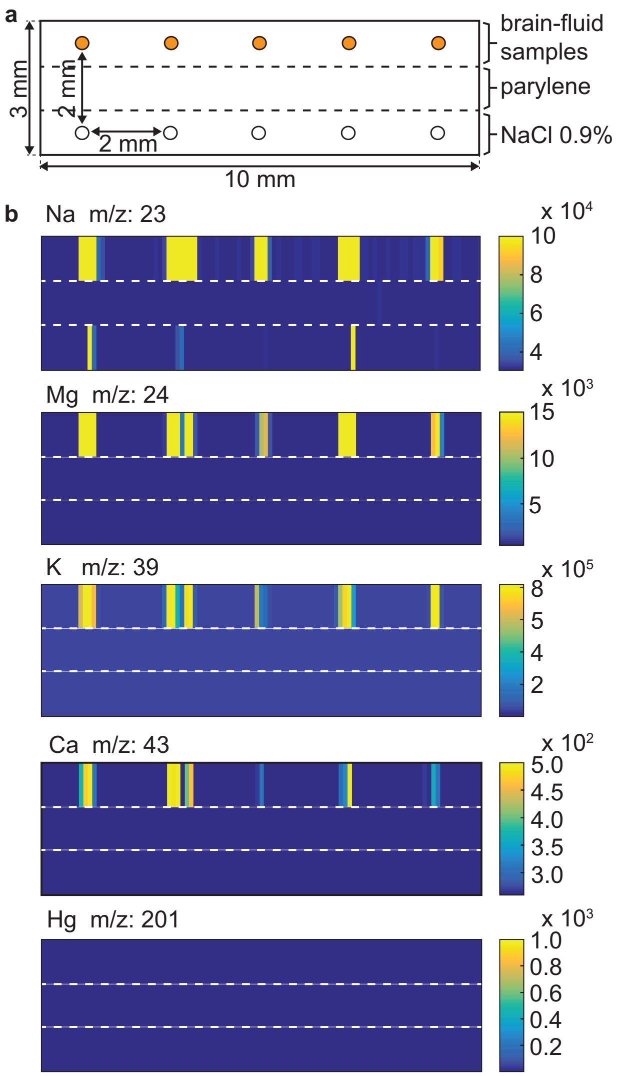

Supplementary Fig.1 Brain-fluid samples imaging after a single laser ablation pass over the brain-fluid samples (a) Samples configuration prior to analysis. The upper section includes the 5 brain-fluid samples (orange spots) while the bottom section comprises 5 control samples of saline solution (0.9 % NaCl, white spots). (b) Relative abundance of the elements Na, Mg, K, Ca and Hg (negative control) found on the brain-fluid samples.

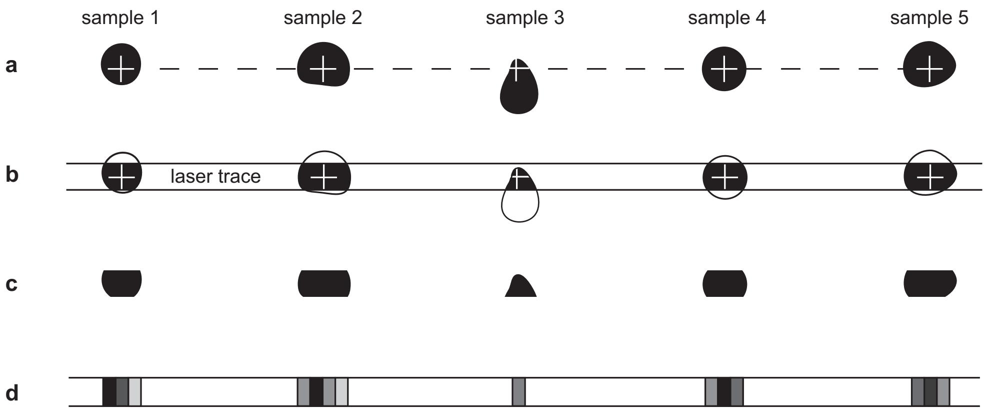

Supplementary Fig.2 Misalignment of the droplet dried spots with respect to the path of the laser beam (a) Dried spots of the brain-fluid samples (black) disposed over the parylene base plate holes (signaled by white crosses). (b) Laser trace over the dried spots. (c) Equivalent area of the dried spots seen by the detection equipment. (d) Final signal readout after analysis (darker means more concentrated).

Method validation

The method trueness and precision were determined with independent Quality Control (QC) samples (volume = $20\,\mathrm{nL}$) prepared at different concentration level. The samples were prepared from a standard multi-element solution (Multi-element 2A, Agilent technologies) diluted at known Cu and Zn concentrations (QC5 = $10\,\mu\mathrm{g\,L^{-1}}$, QC4 = $5\,\mu\mathrm{g\,L^{-1}}$, QC3 = $1\,\mu\mathrm{g\,L^{-1}}$, QC2 = $0.1\,\mu\mathrm{g\,L^{-1}}$, QC1 =$0.05\,\mu\mathrm{g\,L^{-1}}$). The prepared samples were distributed on a parylene base plate in a similar manner as presented in section 2.4.2. Each QC spot was laser ablated in multiple shifted passes and analyzed using an ICP-MS equipment with the same parameters than provided in section 2.4.3. Supplementary Fig. 3 provides the spatial relative abundance of the prepared samples (linear interpolation of 3rd degree was applied on intensity plots). For each QC, four samples were used to determine the method trueness while two samples were used as calibrators (CAL1 and CAL2). The method trueness was determined by computing the percentage difference between the measured concentration and the theoretical concentration which indicates systematic error. The precision, indicating random errors, was assessed by calculating the relative standard deviation in concentration of 4 repeated QCs. The values found are reported in Supplementary Table 1. The method trueness is situated between 92.6 % and 107.0 % while the precision remains under 18.4 % (at QC1). The method linearity was checked by fitting the back-calculated QCs concentrations as a function of the nominal concentration values using the linear regression model based on the leastsquares method. With coefficients of determination (r^2) above 0.992, the method was demonstrated to be linear for Cu and Zn in the evaluated concentration range. The method limit of quantification (LOQ), defined as the lowest concentration of the quality control points with a trueness and precision under 20 % and a signal intensity at least ten times higher than the signal blank (parylene substrate), was demonstrated to be $0.05\,\mu\mathrm{g\,L^{-1}}$.

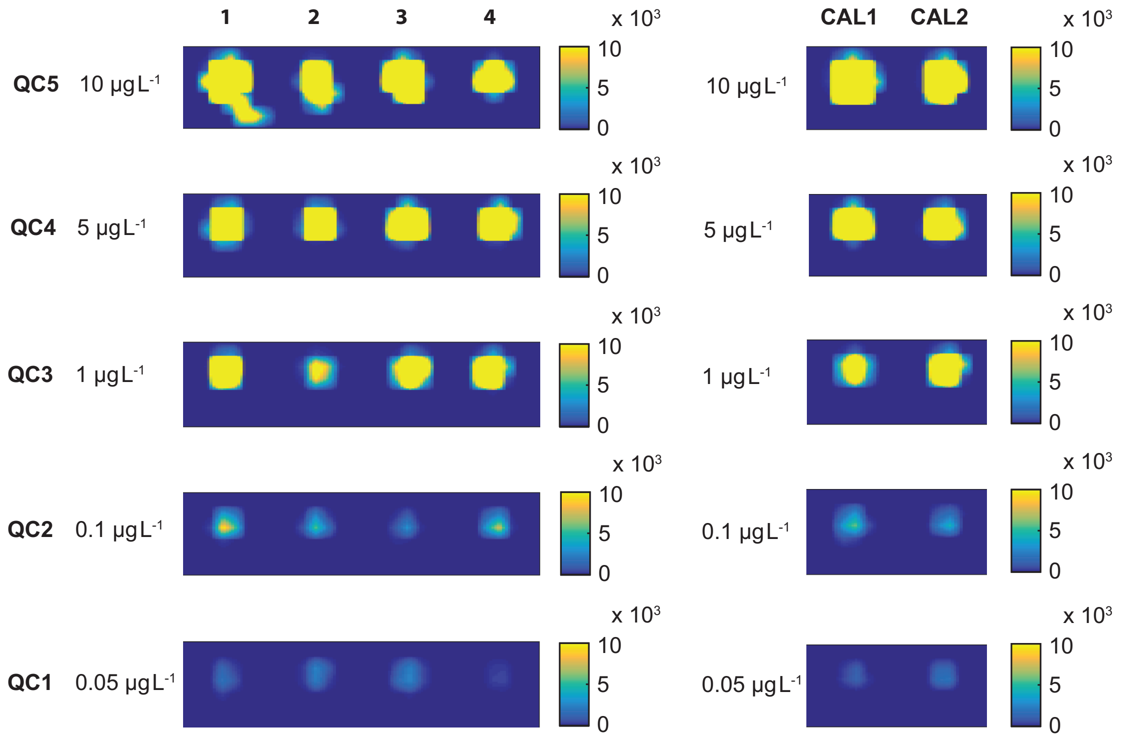

Supplementary Fig.3 Spatial relative abundance of the the Quality Control (QC) samples used for the validation of the analytic method. Four Independent samples of each QC were used to determine the method trueness while two samples were used as calibrators (CAL1 and CAL2).

Supplementary Table 1 Validation data

Element	Nominal concentration ($\mu g L^{-1}$)	Found concentration ($\mu g L^{-1}$)	Trueness (%)	Precision (%)
Cu	10	10.1	101.4	12.6
	5	5.1	102.0	10.6
	1	1.1	107.0	11.1
	0.1	0.10	104.0	12.6
	0.05	0.048	96.5	13.0
Zn	10	9.7	97.3	3.9
	5	5.0	101.0	8.4
	1	1.0	96.3	9.6
	0.1	0.10	99.3	13.2
	0.05	0.046	92.6	18.4

Droplets interface

Supplementary Fig. 2 provides a comparison between the PFD-liquid interface against the inner capillary wall surface when the collected liquid is a water sample colored in red (food dye) (a) and a brain-fluid sample (b). Prior to execution of both experiments the inner wall of the capillary was gas-treated with TMCS (Chlorotrimethylsilane, Sigma-Aldrich) to render the glass surface hydrophobic. Supplementary Fig. 2 (a) shows the PFD-water interface after the collection of water (dyed in red) as part of an experiment performed *ex vivo*. As expected, the PFD demonstrates a high affinity with the inner wall. The value of the contact angle β_1 is $54°$. Supplementary Fig. 2 (b) shows the PFD-water interface after the collection of brain-fluid samples performed *in vivo*. The value of the contact angle β_2 is here $135°$ which demonstrates the surface returned to a hydrophilic state.

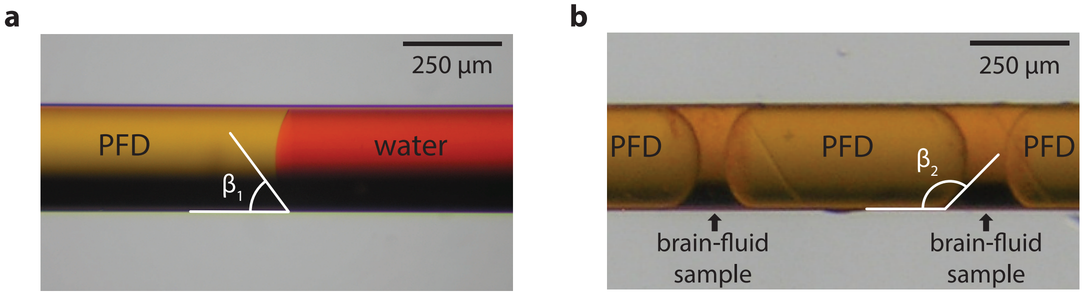

Supplementary Fig.4 Capillary inner surface properties modification after the passage of brain-fluid samples (a) Detail of the PFD-water interface in the collection capillary after the sampling of red-colored water as part of an experiment performed *ex vivo*. The interface angle β_1 equals $54°$. (b) The PFD-brain-fluid interface angle after the collection of samples *in vivo* is much larger with $\beta_2 = 135°$.
